# A Possible Geographic Origin of Endemic Hepatitis C Virus 6a in Hong Kong: Evidences for the Association with Vietnamese Immigration

**DOI:** 10.1371/journal.pone.0024889

**Published:** 2011-09-13

**Authors:** Xiaoming Zhou, Paul K. S. Chan, John S. Tam, Julian W. Tang

**Affiliations:** 1 Department of Epidemiology, Shanghai Public Health Clinical Center, Fudan University, Shanghai, China; 2 Department of Microbiology, Prince of Wales Hospital, The Chinese University of Hong Kong, Shatin, Hong Kong Special Administrative Region, China; 3 World Health Organization, Geneva, Switzerland; 4 Department of Laboratory Medicine, National University Hospital, Singapore; Singapore Institute for Clinical Sciences, Singapore

## Abstract

**Background:**

Hepatitis C virus (HCV) 6a accounts for 23.6% of all HCV infections of the general population and 58.5% of intravenous drug users in Hong Kong. However, the geographical origin of this highly predominant HCV subgenotype is largely unknown. This study explores a hypothesis for one possible transmission route of HCV 6a to Hong Kong.

**Methods:**

NS5A sequences derived from 26 HCV 6a samples were chosen from a five year period (1999–2004) from epidemiologically unrelated patients from Hong Kong. Partial-NS5A sequences (513-bp from nt 6728 to 7240) were adopted for Bayesian coalescent analysis to reconstruct the evolutionary history of HCV infections in Hong Kong using the BEAST v1.3 program. A rooted phylogenetic tree was drawn for these sequences by alignment with reference Vietnamese sequences. Demographic data were accessed from “The Statistic Yearbooks of Hong Kong”.

**Results:**

Bayesian coalescent analysis showed that the rapid increase in 6a infections, which had increased more than 90-fold in Hong Kong from 1986 to 1994 correlated to two peaks of Vietnamese immigration to Hong Kong from 1978 to 1997. The second peak, which occurred from 1987 through 1997, overlapped with the rapid increase of HCV 6a occurrence in Hong Kong. Phylogenetic analyses have further revealed that HCV 6a strains from Vietnam may be ancestral to Hong Kong counterparts.

**Conclusions:**

The high predominance of HCV 6a infections in Hong Kong was possibly associated with Vietnamese immigration during 1987–1997.

## Introduction

Hepatitis C Virus (HCV) is associated with chronic human hepatic diseases which may develop to hepatic cirrhosis or hepatocellular carcinoma [1]. Six major HCV genotypes and more than 18 subgenotypes are currently known [2]. These diversified HCV genotypes often show restricted geographic distributions [3].

HCV 1b and HCV 6a are the two most prevalent subgenotypes distributed throughout Hong Kong [4]. HCV 1b is also established in China, Japan, the United States and Europe [5], [6]. Although HCV 6a is found predominantly in Hong Kong [4], [7], it is not clear whether Hong Kong is the origin of HCV 6a. Residents of Hong Kong are primarily Chinese with a substantial population of British. In China, HCV 2a and 1b were found to be the two most predominant HCV genotypes, which account for more than 95% of infections [8]. In the United Kingdom, HCV 1a, 1b and 3a are the predominant genotypes [9]. It is, therefore, highly unlikely that the HCV genotypes from these two countries are linked to HCV 6a infections in Hong Kong, since HCV 6a was rarely found in China or the UK.

HCV 6a was also detected in Southeast Asia including the countries of Vietnam, Burma and Thailand [10]. It accounted for 37.1% HCV positive samples from blood donors, to be the most predominant genotype in Vietnam [11]. HCV 1a and 1b accounted for other 30.0% and 17.1% positive samples [11]. It is worthwhile to explore the possible relationship of HCV 6a transmission among these areas. Migration of a large number of Vietnamese to Hong Kong occurred from the late 1970s to the 1990s. In this study, we explored the hypothesis that HCV 6a was transmitted from Vietnam to Hong Kong.

NS5A and NS5B are two HCV encoding region for nonstructural protein 5A and 5B. They all can be used for deduct the transmission relationship between multiple HCV isolates without great difference. Their evolutionary history can be postulated from accumulation of mutations maintained in encoded gene sequences [12]. Since there were less HCV 6a sequences can be found from GenBank ever, it had caused great difficulty to this study. Thus, a group of NS5A sequences available for currently collected HCV 6a samples from Hong Kong were used for Bayesian coalescent study to infer historic outbreak events [13]–[17], and another group of NS5B sequences which was just available for samples from both Vietnam source and Hong Kong source were used for reconstruction of an evolutionary phylogenetic tree to infer ancestral strains.

## Materials and Methods

### Sequence Samples

HCV 6a samples used for Bayesian coalescent analysis were collected from chronic hepatitis patients from 1999 to 2004. All patients submitted written consent to take part in tests for this study. Ethical approval was granted by the local Joint Chinese University of Hong Kong-New Territories East Cluster Clinical Research Ethics Committee (ref CRE-2006.405) from where the patients were recruited. All patients were Chinese from Hong Kong. There was no obvious direct epidemiological linkage of these patients, such as familial or cluster infection relationships. Twenty-six HCV 6a samples had been sequenced from the 513-bp NS5A region from nt 6728 to 7240 (EUHK2, GenBank accession no: Y12083). Their sequences were deposited into GenBank (AY859526, DQ525424-DQ525434 and DQ480512- DQ480524).

### Bayesian Coalescent Analysis

Bayesian coalescent analysis was performed using the Bayesian Evolutionary Analysis Sampling Trees program (BEAST, version v1.2.1, http://evolve.zoo.ox.ac.uk/Beast/). This program applies an algorithm of Markov-Chain-Monte-Carlo (MCMC) chain estimated Bayesian inference. The sequences for analysis were aligned using the Clustal-X program (version 1.83, http://www.clustal.org/). Codon-position partitioned Hasegawa-Kishino-Yano model was used to calculate the sequence distances. MCMC-chain length was set to 5×10^6^ repeats to reach a sufficient effective sample size (ESS) of more than 100 for the analysis. A constant molecular-clock was assumed for the evolution of HCV and was calculated from heterochronic sequences using BEAST. The “skyline plot” method was used to reconstruct the demographic history of HCV infections with an assumption that the viral transmission parameters remain constant through time. The BEAST results were further analyzed and demonstrated using the MCMC Trace File Analyzer program (Tracer, version 1.3.1, http://evolve.zoo.ox.ac.uk/Beast/).

### Number of Vietnamese Boat People Entering Hong Kong

The population data were collected from yearbooks of Hong Kong edited by the Information Service Department, Hong Kong Government, in the chapter titled “population and immigration” [18].

### Statistics

The association of the postulated increase of HCV 6a with the population increase of Vietnamese Boat People remained in Hong Kong was carried out by using a logistic regression model.

### Phylogenetic Analysis

All of the 17 HCV 6a NS5B sequences isolated from Hong Kong and all of the 16 HCV 6a NS5B sequences isolated from Vietnam were retrieved from GenBank. Their common 329-bp sequences (8245 to 8593, refer to EUHK2) were used for the phylogenetic analysis. HCV 1a reference strain HCV-H (GenBank accession no: M67463) was included as an outgroup reference for the analysis.

The best-fitting evolutionary model was determined using ModelTest (version 3.06, http://www.cbs.dtu.dk/courses/PR/Modeltest3.06.pdf). A maximum-likelihood phylogenetic tree was constructed with the codon-position partitioned model by using PAUP* (version 4 beta10, http://paup.csit.fsu.edu/downl.html). Bootstrap value was calculated in 1000 replicates.

## Results

### Endemic of HCV 6a Infections

**Figure 1:**
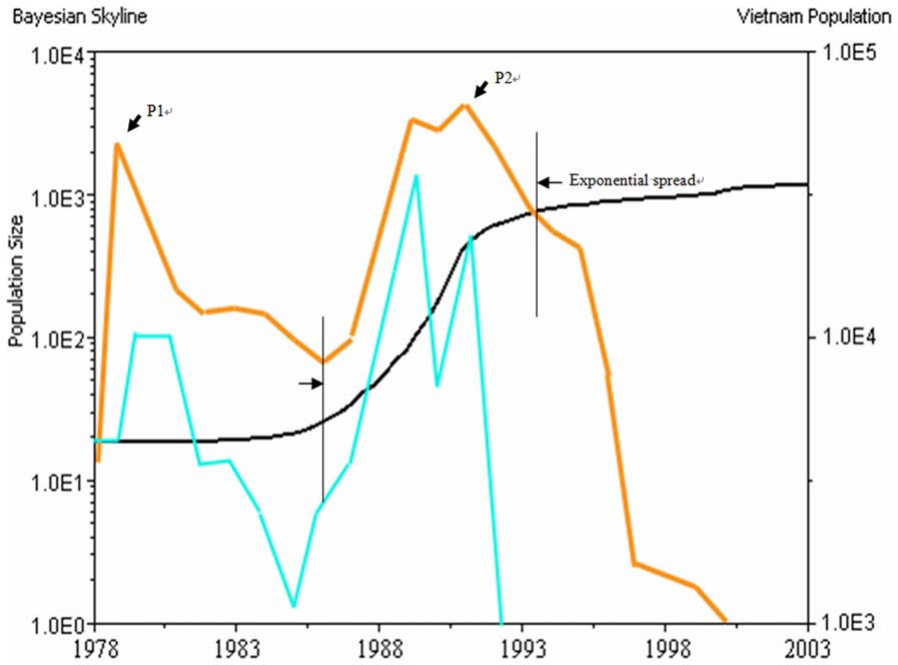
Endemic History of Hepatitis C Virus 6a in Hong Kong. Black line: increases in HCV 6a population estimated using BEAST. Carmine line: population of Vietnamese Boat People remaining in Hong Kong. Blue line: new arrival of Vietnamese to Hong Kong. X-axis: year. Y-axis: *left*: estimated HCV 6a population size, *right*: Vietnamese population. P1: first peak of Vietnamese Boat people arrival to Hong Kong (1979). P2: second peak of Vietnamese Boat people arrival to Hong Kong (1992). Exponential spread: estimated HCV 6a exponential increase period (1986–1994) by BEAST inference.

Transition of constant growth to exponential growth of HCV 6a occurred in 1986 and lasted until 1994 (Figure 1). HCV 6a infections rapidly increased from a population of 1.1×10^1^ to 1.0×10^3^ during this period. The divergence time of the most recent common ancestor (MRCA) of these 26 HCV 6a samples was estimated to1951, 53.4 years (SD 2.9 years) earlier than the most recent date for samples collected in 2004. The rates of evolution at three codon positions were different. At the first codon-position, the rate was 8.3×10^−4^ substitutions/site/yr (SD 3.5×10^−5^). At the second codon position, the rate was 3.8×10^−4^ substitutions/site/yr (SD 1.6×10^−5^). At the third codon position, the rate was 4.0×10^−3^ substitutions/site/yr (SD 1.7×10^−4^) (Table 1).

**Table 1:** Bayesian Coalescent Analysis for Hepatitis C Virus 6a.

	HCV 6a
Number of Samples	26
Time Interval	1999–2004
Mean Likelihood	−2488.6
Effective Sample Size	203
Diversion Time of MRCA	1951 (SD 2.9 years)
Exponential Spread Time	1986–1994
Evolutionary Rate in	
First Codon Position	8.29×10^−4^ (SD 3.5×10^−5^)
Second Codon Position	3.80×10^−4^ (SD 1.6×10^−5^)
Third Codon Position	3.97×10^−3^ (SD 1.7×10^−4^)
MCMC chain Length	5×10^6^

Evolutionary rates are represented in a unit of substitution/site/year. MRCA: most recent common ancestor.

### Vietnamese Boat People

The historic arrival of Vietnamese Boat People began in 1978 and ended in 1997. At that time, a lot of Vietnamese had flooded into Hong Kong seeking resident status. This event occurred during two major periods. The first period was from 1978 to 1982, when the Vietnamese migration was due to political reasons. The second period occurred from 1987 to 1997 (Figure 1), when the Vietnamese migration was due to economic reasons. Most Vietnamese were eventually deported to other countries or repatriated to Vietnam. However, many remained in Hong Kong for extended periods, where most were living in crowded conditions in detention camps during their stay. This type of living condition is usually an important factor contributing to disease outbreaks. The yearly populations of Vietnamese remaining in Hong Kong are shown in Figure 1.

### Association of HCV 6a Endemic Increase with Vietnamese Population

A logistic regression model associated the increase of postulated endemic HCV 6a cases with the size of Vietnamese Boat People (VBP) remained in Hong Kong during time period of 1986–1996 in each year by following formula (R^2^ = 0.6300, *p* = 0.0035):
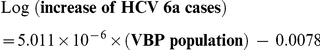



### Phylogenetic Analysis of NS5B Sequences

**Figure 2:**
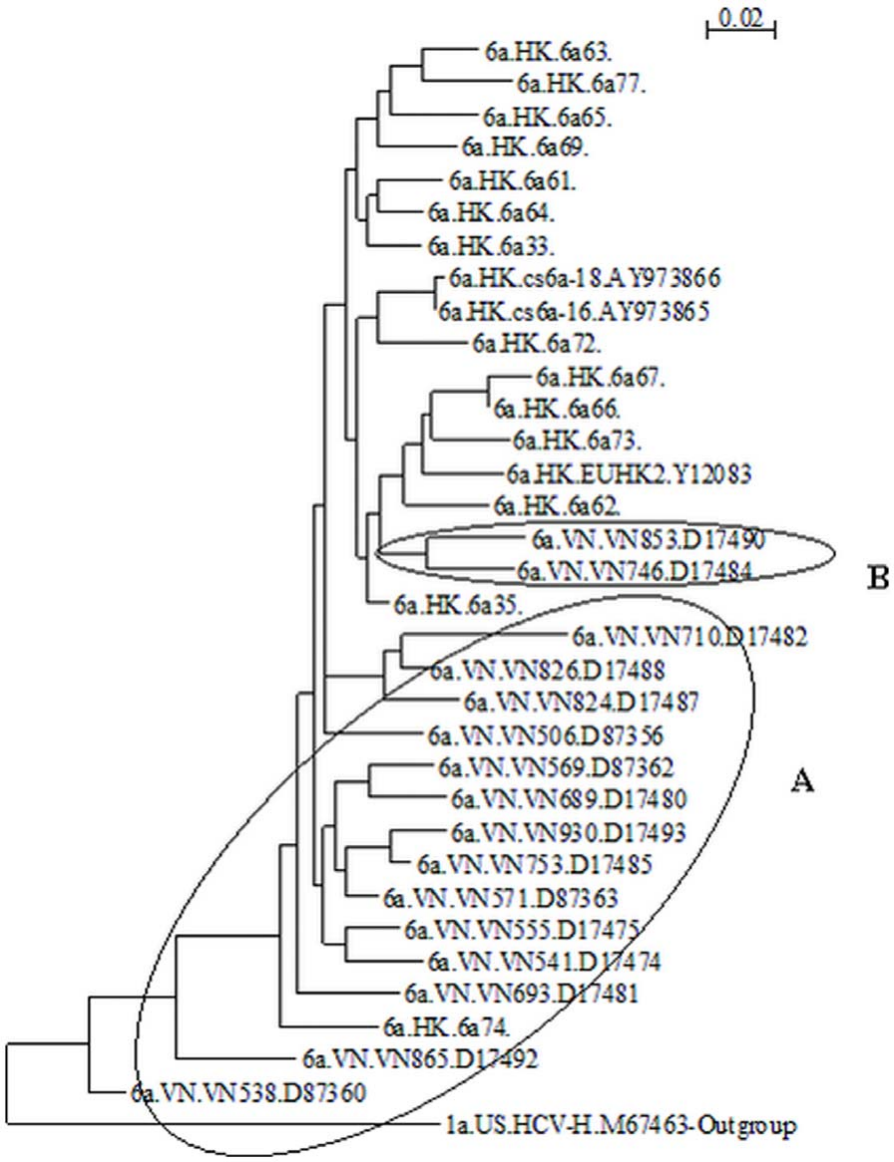
Evolutionary Relationship of Hepatitis C Virus 6a Isolates. HCV sequences were denoted in the format “subgenotype.country.strain. GenBank accession number”. VN: Vietnam, HK: Hong Kong, US: United States. “Outgroup” was labeled for 1a outgroup strain HCV-H. Ellipse “A” encloses those Vietnamese HCV 6a strains (except 6a.HK.6a74) that are located more closely to the origin of evolution. Ellipse “B” encloses two Vietnamese strains that were located parallel to Hong Kong strains. “6a.HK.6ann” denoted samples that were sequenced in this study.

The best evolutionary model for these 33 HCV6a 329-bp partial-NS5B sequences was determined by Modeltest using the General-Time-Reversible (GTR) model. A maximum-likelihood phylogenetic tree was constructed by the codon-position partitioned GTR model. Within this tree, 14 Vietnamese strains were located near the evolutionary origin defined by the outgroup sequence of HCV-H. All 16 Hong Kong strains (except 6a74) were located downstream of the Vietnamese strains. Two Vietnamese strains (VN853 and VN746) were located directly downstream of Hong Kong strain 6a35, but directly upstream of five Hong Kong strains and parallel with ten Hong Kong strains (Figure 2).

## Discussion

Coalescent theory was developed to extract historic population information from currently sampled sequences [17]. In this study, heterochronic sequences across an interval of five years were used for Bayesian coalescent analysis to estimate the evolutionary history of HCV6a in Hong Kong [14], [15].

In this study, a fixed molecular-clock model was assumed for the Bayesian coalescent analysis to limit number of parameters involved in the model estimation process. A previous study investigating the evolution of HIV-1 group O showed that a fixed molecular-clock model will not generate substantial differences compared to a relaxed molecular-clock model (which involved in more number of unnecessary estimated parameters) in estimating the demographic information, when sequences were in evolutionary close lineages [19], [20]. This is just the case in our study. HCV sequences that share homologies of more than 90% were used for analysis.

Evolutionary rates for three codon positions were estimated from sample associated date information. The evolutionary rate of synonymous positions was significantly larger than the rate of the non-synonymous positions of HCV 6a (4.0×10^−3^ *vs* 3.8×10^−4^ substitutions/site/yr, respectively, P<0.01). Hence, a codon-position partitioned model was applied in Bayesian coalescent analysis for a better estimation of the chronological history of HCV infection.

The estimated evolutionary rates in three codon-positions for HCV 6a and for HCV 1b were similar. Their differences were within one single SD. The average evolutionary rate in all positions for HCV 6a was estimated at 4.6×10^−4^ substitutions/site/yr (SD 1.4×10^−4^), with less than one SD difference from the rate of 5.8×10^−4^ estimated previously for HCV 1b [21]. These observations showed that molecular clock rates of our HCV samples were not significantly different from the molecular clock rates of previously analyzed samples.

A time for the diversification of the MRCA strain of these 26 HCV 6a samples was estimated to be the year 1951. An exponential growth period of HCV 6a infections occurred from 1986 to 1994 as revealed by the Bayesian coalescent analysis. This period overlaps with the second peak of Vietnamese Boat People flooding into Hong Kong in 1987–1996, but not the first period in 1978–1982.

To examine the possible transmission direction of HCV 6a between Vietnam and Hong Kong, we constructed a rooted phylogenetic tree consisting of HCV 6a sequences isolated from Vietnam and Hong Kong (Figure 2). HCV-H (Genotype 1a) was used as an outgroup reference for defining the evolutionary origin of the phylogenetic tree. This tree showed that most Vietnamese strains were located in ancestral lineages upstream to most Hong Kong strains. The direction of evolution was obviously from Vietnamese strains to Hong Kong strains.

In conclusion, our results suggested that HCV 6a spreading in Hong Kong was possibly correlated with the arrival of Vietnamese Boat People. A rooted phylogenetic tree showed that Vietnam HCV 6a strains were possibly the ancestors of Hong Kong strains. More evidence is needed to firmly establish an epidemiological linkage of HCV 6a Vietnam strains and Hong Kong strains.

HCV 6a has also been isolated from other southeastern Asian countries. However, available sequence data are very limited. Most HCV 6a sequences in GenBank were from genomic regions of 5′-UTRs, cores and E1-E2-HVRs. These regions are not suitable for evolutionary analysis due to their extreme conservation or hypervariability. More HCV 6a sequences from the structural regions and from different countries are required to elucidate the evolutionary relationship of HCV 6a in Southeast Asia.
